# Quality of the Evidence Supporting the Role of Acupuncture for Stable Angina Pectoris: An Umbrella Review of Systematic Reviews

**DOI:** 10.3389/fcvm.2021.732144

**Published:** 2021-09-30

**Authors:** Min Shen, Jinke Huang, Tao Qiu

**Affiliations:** ^1^Department of Neurology, The First Affiliated Hospital of Zhejiang Chinese Medical University, Hangzhou, China; ^2^Department of Neurology, The First Affiliated Rehabilitation Hospital of Zhejiang Chinese Medical University, Hangzhou, China; ^3^Xiyuan Hospital, China Academy of Chinese Medical Science, Beijing, China

**Keywords:** acupuncture, stable angina pectoris, AMSTAR 2, grade, overview

## Abstract

**Background:** To systematically appraise and synthesize evidence, we conducted an overview of systematic reviews/meta-analyses (SRs/MAs) on acupuncture for stable angina pectoris (SAP).

**Methods:** Eight databases were searched for SRs/MAs of acupuncture on SAP. The methodological quality, reporting quality, and evidence quality were evaluated by Assessing the Methodological Quality of Systematic Reviews 2 (AMSTAR-2), the Preferred Reporting Items for Systematic Reviews and Meta-Analyses (PRISMA) checklist, and the Grading of Recommendations Assessment, Development and Evaluation (GRADE) system, respectively.

**Results:** A total of seven published SRs/MAs met the inclusion criteria. According to the evaluation results of AMSTAR-2, two studies were considered as of moderate quality; the remaining five were considered as of very low quality. According to the evaluation results of the PRISMA checklist, only one study reported the checklist in its entirety, while others had reporting deficiencies. According to GRADE, a total of 18 outcome indicators extracted from the included studies were evaluated. The evidence quality was very low in three, low in three, moderate in eight, and high in four.

**Conclusion:** Acupuncture may be beneficial for SAP from the currently published evidence. However, this conclusion must be interpreted cautiously due to the generally low methodological quality, reporting quality, and evidence quality of the included studies. More rigorous, more standardized and comprehensive SRs/MAs are needed to provide strong evidence for convincing conclusions.

## Introduction

Stable angina pectoris (SAP) is a highly prevalent ischemic cardiovascular disease characterized by left anterior chest pain or discomfort in adjacent areas (1). SAP has become a public health concern worldwide, which affects more than 560,000 individuals per year in America (2). In most European countries, the prevalence of SAP is estimated to be 20,000–40,000 people per million (3). In China, the incidence of SAP is estimated to be 2.4% in men and 3.2% in women (4). Antiplatelet drugs, nitrates, statins, and β-receptor blockers as anti-ischemic treatments are recommended by guidelines as conventional medication (CM) for SAP (5). Despite optimal medical treatment, recurrent angina pectoris is not rare. Thus, patients with SAP resort to complementary and alternative medicine for symptom relief.

Acupuncture, as an important component of traditional Chinese medicine, has been reported to treat cardiovascular diseases, including heart failure (6) and angina (7). According to the reports of published systematic reviews/meta-analyses (SRs/MAs), acupuncture may benefit patients with SAP (8–14). However, the conclusions of these studies regarding the effectiveness of acupuncture in relieving angina symptoms or reducing angina frequency in patients with SAP are not always consistent. To systematically appraise and synthesize these results, we conducted an overview of SRs/MAs. This overview also intended to identify the deficiencies and provide recommendations for achieving high-quality SRs/MAs.

## Methods

### Study Registration

We have registered this overview under the PROSPERO registry (CRD42020188107). The methodology followed the guidelines stated in the Cochrane Handbook, the Preferred Reporting Items for Systematic Reviews and Meta-Analyses (PRISMA) checklist (15), and some high-quality methodological overviews (16, 17).

### Criteria for Inclusion

#### Type of Study

This overview only included SRs/MAs of randomized controlled trials (RCTs) on acupuncture for SAP.

#### Type of Subjects

Subjects diagnosed with SAP according to national or international criteria, regardless of age, race, or sex, were included.

#### Type of Interventions

The intervention method in the experimental group was acupuncture or acupuncture plus conventional medication (e.g., antiplatelet drugs, statins, β-receptor blockers, and nitrates). The intervention method in the control group was CM.

#### Types of Outcome Measurements

The following are the outcome measurements: frequency, intensity of angina, number of patients with changes on the electrocardiogramme (ECG), effective rate, adverse events, and nitroglycerin use.

### Search Methods for Identification of Studies

PubMed, Cochrane Library, Embase, Web of Science, China National Knowledge Infrastructure, Wanfang database, Chinese Biomedical Literature database, and the Chinese Science Journals Database were searched from their inception to December 2020. The following keywords were used in the search: angina pectoris, acupuncture, systematic review, and meta-analysis. The search strategy for PubMed is shown in Table 1.

**Table 1:** The search strategy for Pubmed.

#1 Angina Pectoris [Mesh]
#2 Angina [Title/Abstract] OR Pectoris[Title/Abstract] OR Stenocardia*[Title/Abstract]
#3 #1 OR #2
#4 Acupuncture [Mesh]
#5 Acupuncture [Title/Abstract] OR Acupuncture Treatment* [Title/Abstract] OR Acupuncture Therapy [Title/Abstract] OR Acupuncture Therapies [Title/Abstract] OR Acupotomy [Title/Abstract] OR Acupotomies [Title/Abstract] OR Dry-needling [Title/Abstract] OR Body-acupuncture [Title/Abstract] OR Electroacupuncture [Title/Abstract] OR Electro-acupuncture [Title/Abstract] OR Auricular Acupuncture [Title/Abstract] OR Warm Needle [Title/Abstract]
#6 #4 OR #5
#7 Meta-Analysis as Topic [Mesh]
#8 Systematic Review* [Title/Abstract] OR Cochrane Review* [Title/Abstract] OR Meta-analysis [Title/Abstract] OR Meta analysis [Title/Abstract] OR Meta-analyses
#9 #7 OR #8
#10 #3 AND #6 AND #9

### Study Selection and Data Extraction

Two independent reviewers assessed the literature for inclusion and abstracted the data based on details. Any disagreements were resolved by consensus through discussion. The following details were extracted: author(s), publication year, simple size, interventions, outcomes relevant to this overview, quality assessment methods, and the summary estimate of the intervention effects.

### Evaluation of the Methodological Quality of the Included Studies

Two independent reviewers assessed the methodological quality of the included studies using Assessing the Methodological Quality of Systematic Reviews 2 (AMSTAR-2) (18). According to the information provided in the included studies, each of the 16 standards of AMSTAR-2 is given a rating of “Yes,” “No,” or “Partial Yes.” Any disagreements were resolved by consensus through discussion.

### Evaluation of the Reporting Quality of the Included Studies

Two independent reviewers assessed the *reporting quality* of the included studies using the PRISMA checklist (15). According to the information provided in the included studies, each of the items in the PRISMA checklist is given a rating of “Yes,” “No,” or “Partial Yes.” Any disagreements were resolved by consensus through discussion.

### Evaluation of the Evidence Quality of the Included Studies

Two reviewers independently assessed the evidence quality using the Grading of Recommendations Assessment, Development, and Evaluation (GRADE) (19). Evaluation of the evidence quality mainly considers the following aspects: risk of bias, inconsistency, indirectness, imprecision, and publication bias. Disagreements were resolved by discussion or consensus with a third-party reviewer.

## Results

### Literature Search

**Figure 1:**
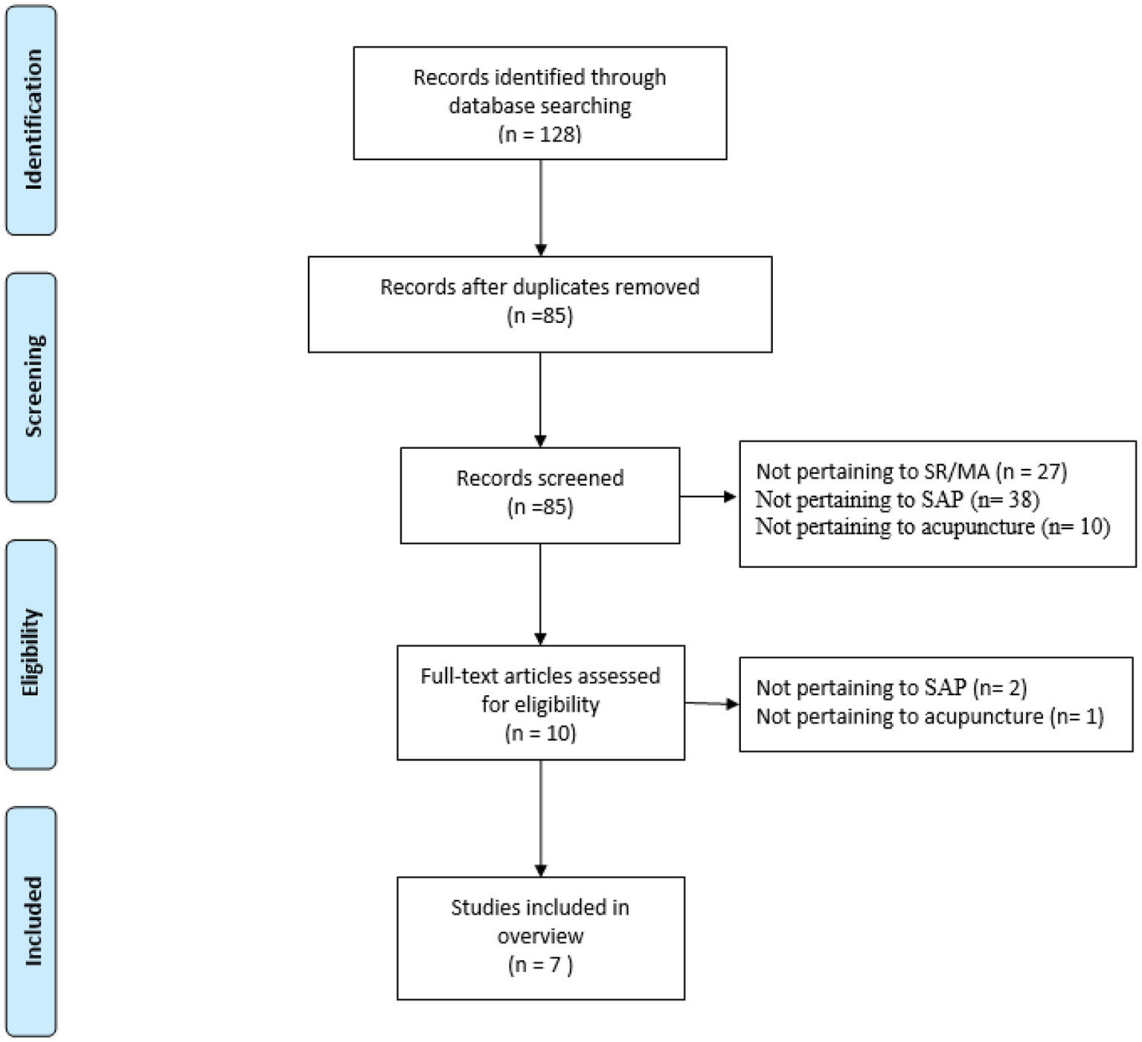
Flow diagram of the review selection process.

The initial database search yielded 128 potentially relevant published studies; 10 publications were retrieved after reviewing the titles and abstracts. Following the review of full texts, seven published SRs/MAs (8–14) met the inclusion criteria. Figure 1 shows details of the flow diagram.

### Description of the Included Studies

All included studies were published between 2012 and 2019. Six of the studies were published in English; the rest are in Chinese (8). The number of trials ranged from 4 to 25, and the sample size ranged from 254 to 2,058. The intervention methods were mostly acupuncture therapy plus CM in the experimental group and CM and sham acupuncture in the control group. For the assessment of methodological quality, six studies used the Cochrane criteria and the rest used the Jadad scale (9). Details are presented in Table 2.

**Table 2:** Characteristics of the studies.

**Author(s), year**	**Country**	**Trials (subjects)**	**Experimental intervention**	**Control intervention**	**Quality assessment**	**Main results**
Zhou et al. (8)	China	4 (254)	AT + CM	CM	Cochrane criteria	The effect of acupuncture plus CM was superior to that of CM alone for the treatment of SAP
Huang et al. (9)	China	24 (1,916)	AT + CM	CM	Jadad	The results support a positive therapeutic effect when using acupuncture as an adjuvant therapy in patients with SAP
Liu et al. (10)	China	12 (974)	AT + CM	CM	Cochrane criteria	Acupuncture may improve angina symptoms and ECG findings in patients with SAP and may be used as an adjunct to this condition
Yang et al. (11)	China	17 (1,516)	AT, AT + CM	Sham AT, CM	Cochrane criteria	Acupuncture may be safe and effective in improving symptoms in patients with SAP. However, angina medication use did not decrease
Yu et al. (12)	China	25 (2,058)	AT, AT + CM	Sham AT, CM	Cochrane criteria	Acupuncture may improve angina symptoms and ECG in patients with SAP. However, large and rigorously designed trials are still needed for further confirmation
Zhang et al. (13)	China	8 (640)	AT, AT + CM	CM	Cochrane criteria	Acupuncture may be helpful for patients with SAP. More clinical trials are still needed to systematically assess the role of acupuncture in SAP
Chen et al. (14)	China	21 (1,899)	AT + CM	CM	Cochrane criteria	Acupuncture combined with CM reduced the occurrence of AMI, relieved angina symptoms, and improved ECG

*AT, acupuncture therapy; CM, conventional medication; SAP, stable angina pectoris; AMI, acute myocardial infarction*.

### Methodological Quality of the Included Studies

Among the included studies, two (11, 12) were rated as of moderate quality; the rest were of very low quality. Items 2 and 7 were rated as of particularly low quality. Only two studies (11, 12) established a prior study protocol. No study provided a complete list of excluded studies with reasons or explained the reasons for the selection of study types. Details are shown in Table 3.

**Table 3:** Results of the AMSTAR-2 assessment.

**Author(s), year**	**AMSTAR-2**																**Quality**
	**I1**	**I2**	**I3**	**I4**	**I5**	**I6**	**I7**	**I8**	**I9**	**I10**	**I11**	**I12**	**I13**	**I14**	**I15**	**I16**	
Zhou et al. (8)	Y	PY	Y	PY	Y	Y	N	Y	Y	Y	Y	Y	Y	Y	Y	Y	VL
Huang et al. (9)	Y	PY	Y	PY	Y	Y	N	Y	Y	Y	Y	Y	Y	Y	N	Y	VL
Liu et al. (10)	Y	PY	Y	PY	Y	Y	N	Y	Y	Y	Y	Y	Y	Y	N	Y	VL
Yang et al. (11)	Y	Y	Y	Y	Y	Y	N	Y	Y	Y	Y	Y	Y	Y	Y	Y	M
Yu et al. (12)	Y	PY	Y	Y	Y	Y	N	Y	Y	Y	Y	Y	Y	Y	Y	Y	M
Zhang et al. (13)	Y	PY	Y	PY	Y	Y	N	Y	Y	N	Y	Y	Y	Y	Y	Y	VL
Chen et al. (14)	Y	PY	Y	Y	Y	Y	N	Y	Y	Y	Y	Y	Y	Y	Y	Y	VL

*AMSTAR-2, Assessing the Methodological Quality of Systematic Reviews 2; Y, yes; PY, partial yes; N, no; VL, very low; L, low; M, moderate; H, high*.

### Reporting Quality of the Included Studies

With the PRISMA checklist, only one study (11) reported all items in the checklist, while others had reporting deficiencies. The checklist items incomplete reported were mainly Q5 (protocol and registration), Q8 (search), Q16 (additional analyses of methods), and Q23 (additional analyses of results). Details are shown in Table 4.

**Table 4:** Results of the PRISMA checklist.

**Section/topic**	**Items**	**Zhou et al. (8)**	**Huang et al. (9)**	**Liu et al. (10)**	**Yang et al. (11)**	**Yu et al. (12)**	**Zhang et al. (13)**	**Chen et al. (14)**	**Compliance (%)**
Title	Q1. Title	Y	Y	Y	Y	Y	Y	Y	100
Abstract	Q2. Structured summary	Y	Y	Y	Y	Y	Y	Y	100
Introduction	Q3. Rationale	Y	Y	Y	Y	Y	Y	Y	100
	Q4. Objectives	Y	Y	Y	Y	Y	Y	Y	100
Methods	Q5. Protocol and registration	N	N	N	Y	N	N	N	14.3
	Q6. Eligibility criteria	Y	Y	Y	Y	Y	Y	Y	100
	Q7. Information sources	Y	Y	Y	Y	Y	Y	Y	100
	Q8. Search	PY	PY	PY	Y	Y	PY	Y	42.9
	Q9. Study selection	Y	Y	Y	Y	Y	Y	Y	100
	Q10. Data collection process	Y	Y	Y	Y	Y	Y	Y	100
	Q11. Data items	Y	Y	Y	Y	Y	Y	Y	100
	Q12. Risk of bias in individual studies	Y	Y	Y	Y	Y	Y	Y	100
	Q13. Summary measures	Y	Y	Y	Y	Y	Y	Y	100
	Q14. Synthesis of results	Y	Y	Y	Y	Y	Y	Y	100
	Q15. Risk of bias across studies	Y	Y	Y	Y	Y	Y	Y	100
	Q16. Additional analyses	Y	Y	Y	Y	Y	N	N	28.6
Results	Q17. Study selection	Y	Y	Y	Y	Y	Y	Y	100
	Q18. Study characteristics	Y	Y	Y	Y	Y	Y	Y	100
	Q19. Risk of bias within studies	Y	Y	Y	Y	Y	Y	Y	100
	Q20. Results of individual studies	Y	Y	Y	Y	Y	Y	Y	100
	Q21. Synthesis of results	Y	Y	Y	Y	Y	Y	Y	100
	Q22. Risk of bias across studies	Y	Y	Y	Y	Y	Y	Y	100
	Q23. Additional analysis	Y	Y	Y	Y	Y	N	N	28.6
Discussion	Q24. Summary of evidence	Y	Y	Y	Y	Y	Y	Y	100
	Q25. Limitations	Y	Y	Y	Y	Y	Y	Y	100
	Q26. Conclusions	Y	Y	Y	Y	Y	Y	Y	100
Funding	Q27. Funding	Y	Y	Y	Y	Y	Y	Y	100

*PRISMA, Preferred Reporting Items for Systematic Reviews and Meta-Analyses; Y, yes; PY, partial yes; N, no*.

### Evidence Quality of the Included Studies

A total of 18 outcome indicators extracted from the included studies were evaluated; the evidence quality was very low in three, low in three, moderate in eight, and high in four. Risk of bias was the most common downgrading factor, followed by imprecision, inconsistency, publication bias, and indirectness. Details are shown in Table 5.

**Table 5:** Results of GRADE assessment.

**Author(s), year**	**Outcomes**	**Trials (subjects)**	**Limitations**	**Inconsistency**	**Indirectness**	**Imprecision**	**Publication bias**	**Relative effect (95% CI)**	**Quality**
Zhou et al. (8)	Effective rate	2 (152)	−1	0	0	−1	−1	OR = 6.01 (1.94–18.66)	VL
Huang et al. (9)	Effective rate	24 (1,916)	−1	0	0	0	0	OR = 2.10 (1.62–2.72)	M
Liu et al. (10)	Angina relief	7 (516)	0	0	0	0	0	RR = 0.35 (0.22–0.55)	H
	ECG improvement	6 (492)	0	0	0	0	0	RR = 0.49 (0.37–0.64)	H
Yang et al. (11)	Angina relief	3 (248)	0	−1	0	−1	0	MD = −4.47 (−6.69 to −2.25)	L
	Nitroglycerin use	2 (226)	0	−1	0	−1	−1	MD = −0.95 (−1.09 to −0.81)	VL
	Average pain intensity	2 (221)	0	−1	0	−1	−1	MD = −0.94 (−2.2 to 0.32)	VL
	ECG improvement	5 (343)	0	−1	0	0	0	RR = 1.25 (1.13–1.37)	M
	Effective rate	6 (448)	0	−1	0	0	0	RR = 1.25 (1.14–1.37)	M
Yu et al. (12)	Angina relief	14 (1,002)	−1	0	0	0	0	RR = 0.33 (0.23–0.47)	M
	ECG improvement	12 (1,035)	−1	0	0	0	0	RR = 0.50 (0.40–0.62)	M
Zhang et al. (13)	Angina relief	8 (672)	0	0	0	0	0	OR = 2.89 (1.87–4.47)	H
	ECG improvement	7 (520)	0	0	0	0	0	OR = 1.83 (1.23–2.71)	H
	Effective rate	2 (122)	−1	0	0	−1	0	OR = 2.13 (0.90–5.07)	L
	Nitroglycerin use	2 (232)	−1	0	0	0	0	MD = −0.44 (−0.64 to −0.24)	M
Chen et al. (14)	Effective rate	10 (752)	−1	0	0	0	0	OR = 0.18 (0.04–0.84)	M
	Angina relief	2 (122)	−1	0	0	−1	0	OR = 4.23 (2.73–6.56)	L
	ECG improvement	10 (747)	−1	0	0	0	0	OR = 2.61 (1.83–3.73)	M

*GRADE, Grading of Recommendations Assessment, Development and Evaluation; OR, odds ratio; RR, relative risk; MD, mean difference; CI, confidence interval; VL, very low; L, low; M, moderate; H, high*.

### Summary of Results of the Included Studies

#### Efficacy of Acupuncture for SAP

The outcome indicators extracted from the included studies are presented in Table 4. Five studies (10–14) reported the outcome indicator for angina relief; meta-analysis revealed a more significant effect of combination therapy than of CM alone. Five studies (10–14) reported the outcome indicator for ECG improvement; meta-analysis showed a more significant effect of combination therapy. Five studies (10–14) reported the outcome indicator for ECG improvement; meta-analysis revealed a more significant effect of combination therapy. Two studies (11, 13) reported the outcome indicator for nitroglycerin use; meta-analysis showed a more significant effect of acupuncture than of CM. Similarly, one study (11) reported the outcome indicator for average pain intensity; the results revealed a more significant effect of combination therapy. Five studies (8, 9, 11, 13, 14) reported the outcome indicator for effective rate; meta-analysis revealed a more significant effect of acupuncture in four studies. However, another study (13) reported no significant difference.

#### Safety of Acupuncture for SAP

Adverse events were mentioned in five studies (10–14), and no adverse effects related to acupuncture were reported.

## Discussion

SRs/MAs are regarded as the gold standard for assessing the effects of an intervention. Despite the strengths of SRs/MAs, bias may arise in the implementation process. An increasing number of SRs/MAs have begun to investigate the effectiveness of acupuncture for SAP. Under the circumstances, to systematically appraise and synthesize these results, we performed this overview.

### Summary of Quality

Through a comprehensive search, seven SRs/MAs on the effectiveness of acupuncture for SAP were identified. According to the results of the AMSTAR-2 assessment, only two of which were regarded as of moderate methodological quality; the rest were of very low methodological quality. Items 2 (protocol registration) and 7 (list of excluded studies) were rated as of particularly low quality. In performing SR/MA, a pre-registration protocol is quite essential, which helps to promote processing transparency and avoid methodological bias (20). In this context, registration platforms, such as PROSPERO, have been highlighted and advocated. Therefore, more attention should be paid to registering the work. Future investigators should register protocols in advance, before the SRs/MAs are carried out. For study selection, all the included studies were selected in duplicate, resolving disagreements by discussion or consensus with a third author. However, none of the studies provided a list of excluded trials, which made it difficult to guarantee the reproducibility of the results (21). Similar to the results of the AMSTAR-2 evaluation, assessment using the PRISMA checklist found that only one study (11) reported the checklist in its entirety, while others had reporting deficiencies. Incomplete reports of checklist items were mainly missed information on protocol and registration, literature search, and additional analyses.

The evidence quality was assessed by the GRADE system. Among the 17 outcome indicators, the evidence quality was very low in three, low in three, moderate in eight, and high in four. Risk of bias was the most common downgrading factor, followed by imprecision, inconsistency, publication bias, and indirectness. Through further analysis, it was found that the limitation in this area was mainly due to the inability of acupuncture therapy to blind the implementer. Thus, there are issues that should be paid attention to. With the development of evidence-based acupuncture, it is hoped that researchers will continue to promote standardization of the techniques and operating procedures of acupuncture in the future in order to provide more high-quality evidence.

Of the included studies, it is worth noting that, although almost all indicated that acupuncture is an effective approach in treating SAP, most authors did not want to draw firm conclusions because of the small sample sizes or the low methodological quality of the included studies. Meanwhile, given that the results assessed in this overview suggest that neither the methodological quality of the included studies nor the evidence quality of the reported outcome indicators is satisfactory, we cannot draw firm conclusions regarding acupuncture for SAP based on the currently published evidence. Therefore, in the future, RCTs with adequate blinding and large simple sizes are still needed to determine whether acupuncture or combination therapies are more effective than other treatments. Furthermore, strict adherence to the Cochrane Handbook for the design and implementation of SRs/MAs needs to be encouraged and advocated in order to be able to provide more convincing evidence.

### Limitations

To the best of our knowledge, this is the first study using the method of overview to systematically appraise and synthesize evidence of acupuncture for SAP. The results of this overview will be beneficial to evidence users and help researchers improve the quality of their studies. However, limitations should be acknowledged, especially the differences involving populations, interventions, comparators, outcomes, and study designs (PICOS). Firstly, all included SRs/MAs mentioned that the included patients were diagnosed with SAP according to national or international standards. However, only one study (12) mentioned the use of the WHO criteria, while other studies did not account for the specific diagnostic criteria used. Moreover, patients were heterogeneous in terms of age, disease duration, and complications. The above heterogeneity from the patient aspect may affect the credibility of the conclusions. Secondly, some studies have looked at the use of acupuncture to combine other interventions on a control group basis, which is easily mixed with the effects of acupuncture. Thus, experimental measures of acupuncture alone or plus a control group may increase credibility. Thirdly, the diversity of the acupuncture treatment protocols has resulted in heterogeneity, including acupoint selection, stimulation methods, and the frequency of treatment. Although the use of some acupoints was concentrated, the overall differences in the acupuncture treatment regimens in the included studies were large. Moreover, the CM used in the control group was different. This may have caused some deviation. Fourthly, the measurement scales used in the RCTs were subjective clinical assessment tools based on the observations of assessors and may not be accurate for the assessment of treatment effects. The use of effective rate or the number of patients with no improvements in ECG also limited the generalization of the results. A fifth limitation is that, although all SRs/MAs were based on RCTs, inadequate reporting of randomization, allocation concealment, blinding, intention-to-treat analysis, and dropout interpretation in most RCTs may have contributed to the risk of bias. Finally, all the included studies in this overview were performed in China, so whether the conclusions of this study can be generalized to other populations remains to be studied further.

## Conclusions

Acupuncture may be beneficial for SAP based on the currently published evidence. However, this conclusion must be interpreted with caution due to the generally low methodological quality, reporting quality, and evidence quality of the included studies. More rigorous, more standardized and comprehensive SRs/MAs are needed to provide strong evidence for convincing conclusions.

## Data Availability Statement

The original contributions presented in the study are included in the article/supplementary material, further inquiries can be directed to the corresponding author/s.

## Author Contributions

MS and JH wrote the original draft. TQ reviewed and edited the manuscript. All authors contributed to the article and approved the submitted version.

## Conflict of Interest

The authors declare that the research was conducted in the absence of any commercial or financial relationships that could be construed as a potential conflict of interest.

## Publisher's Note

All claims expressed in this article are solely those of the authors and do not necessarily represent those of their affiliated organizations, or those of the publisher, the editors and the reviewers. Any product that may be evaluated in this article, or claim that may be made by its manufacturer, is not guaranteed or endorsed by the publisher.

## Abbreviations

SR: systemic review
SAP: stable angina pectoris
MA: meta-analysis
AMSTAR-2: Assessing the Methodological Quality of Systematic Reviews 2
PRISMA: Preferred Reporting Items for Systematic Reviews and Meta-Analyses
GRADE: Grading of Recommendations Assessment Development and Evaluation
RCTs: randomized controlled trials
CM: conventional medication
ECG: electrocardiogram
PICOS: populations, interventions, comparators, outcomes and study designs.
